# Reproductive Strategies and Romantic Love in Early Modern Europe

**DOI:** 10.1007/s10508-023-02759-4

**Published:** 2023-12-26

**Authors:** Mauricio de Jesus Dias Martins, Nicolas Baumard

**Affiliations:** 1 Département d’Etudes Cognitives, Institut Jean Nicod, École Normale Supérieure, École des Hautes Études en Sciences Sociales, Centre National de la Recherche Scientifique, Paris Sciences and Lettres Research University, 75005 Paris, France; 2 Neurology Department, Max Planck Institute for Human Cognitive and Brain Sciences, 04103 Leipzig, Germany; 3 SCAN-Unit, Department of Cognition, Emotion, and Methods in Psychology, Faculty of Psychology, University of Vienna, 1090 Vienna, Austria

**Keywords:** Passion, Tenderness, Living standards, Nuptial rates, Digital humanities, Early Modern Period

## Abstract

In Western Europe, the Early Modern Period is characterized by the rise of tenderness in romantic relationships and the emergence of companionate marriage. Despite a long research tradition, the origins of these social changes remain elusive. In this paper, we build on recent advances in behavioral sciences, showing that romantic emotional investment, which is more culturally variable than sexual attraction, enhances the cohesion of long-term relationships and increases investment in children. Importantly, this long-term strategy is considered especially advantageous when living standards are high. Here, we investigate the relationship between living standards, the emotional components of love expressed in fiction work, and behavioral outcomes related to pair bonding, such as nuptial and fertility rates. We developed natural language processing measures of “emotional investment” (tenderness) and “attraction” (passion) and computed romantic love in English plays (*N* = 847) as a ratio between the two. We found that living standards generally predicted and temporally preceded variations of romantic love in the Early Modern Period. Furthermore, romantic love preceded an increase in nuptial rates and a decrease in births per marriage. This suggests that increasing living standards in the Early Modern Period may have contributed to the emergence of modern romantic culture.

**Supplementary Information:**

The online version contains supplementary material available at 10.1007/s10508-023-02759-4.

## Introduction

In modern industrial societies, in both Western and non-Western cultures, mutual affection and emotional attachment are considered essential components of romantic relationships and conjugal arrangements (Coontz, 2006; Giddens, 2013). This phenomenon is relatively recent in human societies (Chagnon, 2012; Coontz, 2006; Goody, 2010; Gregor, 2008). For most of history, marriages were based on economic and social considerations (Duby, 1978; Klapisch-Zuber, 1987; Wiesner-Hanks, 2000). However, mutual affection and emotional attachment started being socially valued during the Early Modern period (1500–1800) in Western Europe (Coontz, 2006; Daumas, 1996; Giddens, 2013; Macfarlane, 1986), China (Lu, 2021; Pan, 2015), Japan (Pan, 2015), and India (Orsini, 2006).

The study of private correspondence, diaries, manuals of good manners, and portraits shows a significant change regarding the importance of love (Ariès, 1982; Coontz, 2006; Daumas, 1996, 2004; Macfarlane, 1986; Stone, 1979). For instance, in letter-writing manuals, the proportion of women’s love letters referring to love increased from 12% in the early seventeenth century to 45% in the early eighteenth century (Daumas, 2004). Similarly, while fewer than 10% of French couples seeking annulments before the 1760s argued that marriage should be based on emotional attachment to be fully valid, by the 1770s, more than 40% thought so (Coontz, 2006).

What are the reasons behind such a change in early modern and modern societies? In human behavioral ecology, romantic love is seen as a “commitment device” that facilitates establishing and maintaining long-term interactions between parents, necessary to provide the significant investment required to rear children (Fletcher et al., 2015). From a behavioral ecology perspective, the importance of pair bonding and mutual affection should thus vary with the importance of parental investment.

One important factor of variation in parental investment is resource availability. Recent empirical work has demonstrated that parental investment is higher when resources are higher across countries and across social classes within the same country (Belsky et al., 1991; Brumbach et al., 2009; Chisholm et al., 2005; Del Giudice, 2020; Nettle, 2010, 2012; Nettle & Cockerill, 2010; Quinlan, 2007; Quinlan & Quinlan, 2007). This is also visible in history. Qualitative surveys demonstrate that across past societies (Greece, Rome, China, Europe, Japan), people tend to pay more attention to children and grant more importance to childhood in periods of higher economic affluence (Cunningham, 1998; Dixon, 1992; Heywood, 2017; MacLehose, 2008; Morewitz et al., 2003; Shih, 2015; Swanson, 1990; Wu, 1995). These observations are often considered to result from adaptive responses to variations in resources or mortality (Del Giudice, 2020; Del Giudice et al., 2015).

The same reasoning predicts that pair bonding and mutual affection should vary with levels of resources. Romantic attachment is higher when resources are higher and more predictable, and across societies, higher parental investment tends to be associated with higher romantic investment and emotional attachment, measured as relationship commitment and satisfaction, quality of communication, and monogamy (Li & Zheng, 2021; Quinlan & Quinlan, 2007). This is also the case in history. In a recent paper, Baumard et al. (2022) reviewed the qualitative literature on cultural history. It showed that, when the data are available (Greece, Rome, Europe, China, Japan), a higher importance of love in literary narratives is associated with more positive attitudes toward children.

From a behavioral ecology perspective, parental investment and romantic attachment should also vary with a range of other traits that are more adaptive when resource availability is higher (Pepper & Nettle, 2017). In line with this idea, several empirical works have shown that people invest more in long-term parenting and pair bonding in more favorable environments (Del Giudice, 2020; Nettle & Frankenhuis, 2019).

In this paper, we tested the idea that the rising importance of mutual affection and emotional attachment during the Early Modern and Modern periods is a contingent response to increased resources and the ensuing changes in reproductive strategies. A priori, this idea fits with the economic evolution of Western Europe at the time. From 1500 to 1800, living standards greatly increased in Western Europe, especially in England, where urbanization increased from 2 to 23% (Bosker et al., 2013), per capita gross domestic product (GDP) doubled from $1000 to $2000 (Broadberry et al., 2015), and labor wages increased from 1 to 3,5 consumption baskets (i.e., the number of people that labor wages could sustain; Humphries & Weisdorf, 2019). Similar trends, though lower and later, were also visible in the rest of Western Europe (Bosker et al., 2013; Broadberry et al., 2015). Interestingly, the Early Modern Period also saw increasing attention paid to children, including in fiction, art, and philosophy (Ariès, 1965; Cunningham, 1998; Fass, 2013; Heywood, 2017; Stone, 1979).

To test this idea, we turned to fiction. Love is one of the most common topics in fiction works (Booker, 2004; Gottschall et al., 2005; Hogan, 2003), and historians have long used these to track the change in mentalities regarding love and tenderness (Daumas, 1996, 2004). For instance, Greiner notes that in the French fiction of the 1580–1620 s, the relationship between the two sexes evolved from an ancient representation of women as passive objects of idolatrous worship to an active role as the subject of desire. In other words, fiction started to describe the feelings and desires of women as active agents in romantic interactions. The vocabulary itself bears the imprints of this change since, at the same time, “reciprocal” and “mutual” became almost obligatory adjectives of “love” and “affection” (Greiner, 2017).

In this paper, we assume that the importance of affection and tenderness in fiction reflects a similar importance in the audience’s psychology. Recent works in psychology and social sciences have demonstrated that fiction reflects people’s priorities and personality traits. For instance, using the Facebook myPersonality Database (*N* = 3.5 million), Nave et al. (2020) have shown that specific genres (e.g., fantasy, romances) are associated with specific personality traits. Studying more fine-grained literature, Dubourg et al. (2023) have also demonstrated that imaginary worlds are consistently preferred by individuals who score higher on openness to experience.

Similar observations have been made for romantic love (Galloway et al., 2015). Recently, Monsjou and Mar (2019) found a strong association between the importance of long-term and mutual affection and the interest in reading romantic fiction. Participants more interested in romantic relationships in fiction were also more likely to (1) be and want to be in a romantic relationship, (2) have longer, more satisfying, and emotionally closer relationships, (3) entertain more romantic beliefs, and (4) have stronger views of how romantic relationships should be. Overall, this research suggests that the production and consumption of romantic fiction can be an index of the importance of romantic attachment within a given culture.

We chose to analyze theater plays (1550–1800, *n* = 847) because these were abundant and popular from the late sixteenth century onward, in contrast with the novel, which really emerged in the middle of the eighteenth century. Our custom dataset is also more suitable than most datasets (e.g., Google ngram) due to its broader coverage of the Early Modern Period and its homogeneity (it only includes works of fiction belonging to the same literary genre). Also, in contrast to other genres, such as the novel or the various genres of poetry, theater plays are uniform over the period because of the dialogue constraints in stage performance. This means that the frequency of words is less sensitive to changes in style. Finally, this method has been successfully used to study the shift from dominant- to trust-based attitudes in the same corpora (Martins & Baumard, 2020).

Previous work has documented the association between the cultural salience of romantic love in fiction and resource availability throughout the longer arch of history (Baumard et al., 2022) . The current work introduces methodological changes to more precisely measure the dynamics in the Early Modern Period. First, our earlier study tested the association between love and resources in the very long term and was thus constrained in terms of economic data and temporal granularity. It often relied on imperfect proxies of economic development like density and urbanization. Because we focus on a more recent period, we can use more accurate measures like wages and GDP per capita. Second, this previous study used a methodology compatible with ancient and recent periods, for which fiction texts are not digitized and/or unavailable in English. This constraint led to using the plot summaries rather than the actual texts, resulting in a much smaller dataset for the most recent periods.

Finally, texts provide richer material than plot summaries. In particular, it is possible to distinguish between different kinds of romantic feelings and to test their validity robustly. The concept of love includes various components (Hatfield & Rapson, 1987; Hendrick & Hendrick, 1986), and its expression ranges from passion and physical attraction (Hatfield et al., 2007) to manifestations of emotional investment and attachment (Schmitt, 2008; Schmitt et al., 2009). Overall, we hypothesize that the rise of living standards in the Early Modern Period is associated with increased expression of the emotional components of romantic love compared to the general expression of sexual desire.

To isolate the specific components of romantic love, following the anthropological literature reviewed above, we measured the frequency of words expressing emotional attachment and tenderness and those focused on sexual desire and passion. Then, we computed a romantic love ratio, which measured the salience of tender love in texts relative to the salience of passionate love (see Supplementary Methods and Table S1). While the salience is not a direct indication of directionality per se (whether tender love is discussed with a positive or negative tone), our proxy of tenderness tends to co-occur with the salience of moral and mental qualities, asceticism, family, friendship, and future orientation. Conversely, the salience of passionate love tends to co-occur with physical qualities, sex, sensuality, and present orientation. This covariation structure provides a measure of validity to our proxy of romantic love (see Method for details).

To assess how the emotional components of love are affected by environmental resources in England, we tested how our measure of romantic love is affected by wages (Humphries & Weisdorf, 2019), per capita GDP (GDPpc) (Broadberry et al., 2015), and life expectancy (Zijdeman & Ribeira da Silva, 2023) across time. We analyzed whether living standards correlate with the cultural expression of romantic love in fiction and whether rising living standards temporally precede an increased expression of romantic love. We used cross-correlation and lag analyses to evaluate the temporal precedence between the time series.

In addition to the relationship between living standards and the cultural salience of romantic love in fiction, we also explored the interaction between these psychological shifts and behavioral changes. If romantic love is a commitment device for long-term bonding and investment in child rearing, then shifts in its cultural expression might also impact sexual behavior, such as nuptial and fertility rates. The relationship between standards of living and fertility is nonlinear. On the one hand, short-term fluctuations in GDPpc are associated with procyclical changes in fertility both in contemporary and pre-industrial societies; on the other hand, secular GDPpc growth seems associated with dramatic drops in fertility rates, especially during the second industrial revolution around 1870 (Chatterjee & Vogl, 2018; Galor, 2005; Sobotka et al., 2011). Traditional explanations for the latter effect range from the increased participation of women in the labor force (Chatterjee & Vogl, 2018) to the rise of demand for human capital for mentally intensive jobs (Galor, 2005). Nuptial and birth rate estimates are available for England for the entire period in this study (de Pleijt, 2018; Wrigley et al., 1997).

In sum, our specific hypotheses were that (1A) the importance given to romantic love in fiction will correlate with living standards (GDP per capita, wages, and life expectancy), and (1B) living standards trends will temporally precede isomorphic variations in the importance of romantic love. Our analysis will be rather exploratory regarding the relationship between love and behavior. We tentatively hypothesize that the importance of romantic love will positively correlate with and temporally precede (2A) an increase in nuptial rates (long-term pair bonding) and (2B) a decrease in fertility rate (investment in fewer offspring). Finally, we tested hypotheses 1A and 1B for France (*n* = 707), for which we had GDPpc data up to 1800 but no other reliable environmental data for that historical period.

## Method

### Text Analysis

As a general approach, we acquired English (1550–1800 CE) and French (1550–1900 CE) theatrical texts from online repositories, preprocessed them using the Python Natural Language Toolkit, and for each text, calculated a romantic love measure as a ratio between the frequency of words related to tenderness vs passion, when they were used in the vicinity of the word “love” (and derivations). Thus, we could capture whether the topic of romantic love was more salient than passionate love or vice versa.

In this paper, we used a bags-of-words approach, wherein we first counted the occurrence of all words related to tender love for each text and the occurrence of all the words related to passionate love. To obtain the appropriate bags of words (BoW) of tender and passionate love and the respective romantic love ratio, we followed a procedure described in Martins and Baumard (2022) aimed at ensuring (1) the spatial and semantic proximity of these bags of words to the topic of love, and (2) the internal and external validity of the subsequent ratios. Crucially, we measured the frequency of words related to tenderness and passion only when they were used in the vicinity of seed words “love,” “lover,” “beloved,” and “loving,” thus ensuring the specificity of our measure to the topic of love. We also used word2vec to ensure the semantic adequacy of our BoW items to the particular historical context and eliminated terms if necessary. Finally, we (1) internally validated the consistency of our measures using a factor analysis and (2) externally validated the romantic love ratio by comparing its variance with related proxies from the well-validated bag-of-words tool Linguistic Inquiry and Word Count (LIWC) (Pennebaker et al., 2015; Piolat et al., 2011). More details about each of these steps are given below.

We then modeled how variation in these ratios was explained by the effects of time and living standards, using per capita GDP (GDPpc), wages, and life expectancy as proxies of the latter. We performed cross-correlation analyses between these love ratios and each proxy of living standards to assess the causal relationship between affluence and romantic love (see Analysis section below). We were also interested in the relationship between love and behavioral changes related to investment in partners and children. The latter was proxied as nuptial rates and birth rates. We also performed cross-correlation and lag analyses between romantic love and these variables.

Hypotheses and methods were preregistered in OSF before the analysis (https://osf.io/wn6fs/?view_only=38517edfcabb48be84ee4afd3103057d). The full analysis was performed only for the English data. Due to limitations in the availability of socioeconomic and demographic data for France, we used the French dataset only to partially replicate the findings concerning the relationship between GDPpc (up to 1800) and romantic love.

### Source Materials

To test our hypotheses, we analyzed the text of theater plays written between 1550 and 1800. These plays were collected from several different repositories. Our original sample of 932 plays was divided into two main periods: (1) The Early Modern Period spanning the years 1550–1660 and (2) the Restoration/eighteenth century period spanning the years 1660–1800. For the Early Modern Period, we collected 324 XML sources from the database https://emed.folger.edu/corpus-search (Brown et al., 2020). These plays were already lemmatized and translated into Modern English. In addition, 38 texts of Shakespeare’s plays were collected from an associated source https://www.folgerdigitaltexts.org/download (Mowat et al., 2016). For the second period, due to the lack of availability of systematic theater repositories, we mined general databases for the Restoration (https://quod.lib.umich.edu/e/eebogroup/) and the eighteenth century (https://quod.lib.umich.edu/e/ecco/). We used custom Python scripts (written in Jupyter notebook) to download all the documents containing at least one of the keywords in the list [“tragedy,” “tragic,” “comedy,” “pastoral,” “drama,” “theater,” “theatrical,” “tragic,” “play,” “farse,” “farce,” “comic”] in the title.

In addition, we excluded documents containing the words “opera” and “musical” in the title. In this study, we focused on theater plays as they were broadly popular throughout 1550–1800, thus ensuring a homogeneous dataset. By using a selective strategy, we focused on cultural artifacts produced in a similar context to fulfill a similar function and for a similar audience. For instance, novels and operas became popular in England later in the eighteenth century and were attended by a different audience. This would introduce noise in our diachronic analysis.

We visually inspected the list of downloaded files and further excluded documents not containing theater plays. The final sample for this period was 570 texts. Python scripts and all text sources are available at https://osf.io/ka3th/?view_only=9d827995280c42a5ab89fa7cbcedac03. For French theater, we collected 1060 text files from the repository http://www.theatre-classique.fr/pages/programmes/PageEdition.php, plays written between 1550 and 1900, and with a genre tag containing the keywords [“comédie,” “tragédie,” “farce,” “pastorale,” “drame,” “parodie,” “proverbe”] and excluding [“ballet,” “musique,” “liturgique”]. We also excluded translations of Greek tragedies.

### Text Preprocessing

The first preprocessing step was removing the prologue, epilogue, and non-spoken text, such as character names and action descriptions. This was done to reduce the noise across plays due to large variability in the availability of prologues and action descriptions in our various datasets. We used XML and TXT parsing tools [e.g., BeautifulSoup (Richardson, 2007) and ElementTree (Schmid, 1994)] and custom Python functions. Scripts for theater text preprocessing and output files are available at https://osf.io/ka3th/?view_only=9d827995280c42a5ab89fa7cbcedac03.

The second step was a standard lowercasing of the text, removal of non-literal characters, expansion of contractions, including archaic contractions (e.g., e’en ➔ even), and finally, word lemmatization using WordNetLemmatizer() (Princeton University, 2010). Scripts and output files are available at https://osf.io/ka3th/?view_only=9d827995280c42a5ab89fa7cbcedac03.

After preprocessing (including only nouns, adjectives, and verbs), the total word count was 4.4 M words for the English data and 2.5 M words for the French data.

### Building the Sets of Search Terms (Bags of Words)

We used a combination of strategies to generate the bags of words associated with the different components of love.

First, we extracted the set of the 1000 most common nouns and adjectives occurring in the vicinity of love-related “seed” words within a window of three words before and after the seed, including only adjectives and nouns. For English, we used “love,” “lover,” “beloved,” and “loving” as seeds. This step allowed us to capture the nouns and adjectives most frequently used within the “spatial” context of love.

Second, we sorted words relevant to love and classified them according to ten broad categories (bags of words) denoting emotional investment or physical attraction/desire. The main categories were passionate feelings (e.g., passion, desire, jealousy) and tender feelings (e.g., tenderness, affection, fondness). However, we measured other relevant categories such as sensuality (e.g., pleasure, breast, bosom); discipline/asceticism (e.g., modest, chastity, innocence); physical qualities (e.g., young, handsome, beautiful); moral qualities (e.g., noble, virtue, judgment); short relationships (e.g., conquest, adventure, affair); long relationships (e.g., marriage, family, oath); other body parts (e.g., hand, face, eye); other mental parts (e.g., sound, mind, reason).

Third, to ensure the semantic adequacy of the chosen terms for each bag of words, we used the word2vec algorithm (Mikolov et al., 2013) and checked if the words within our selection had the desired meaning in the corpus. Word2vec is an algorithm that takes all the sentences in the corpus as input. It automatically associates each word in the sentence to a vector (a set of coordinates) in a high-dimensional space, which describes the context in which each word is used. For instance, if two words tend to be used in similar sentences—e.g., “I like to ride a pony” and “I like to ride a horse”—they will be closer in the semantic space. Using this procedure, we trained a vector space with our corpus and computed the set of 10 words most similar to each word in each bag of words. For example, the 10 most similar words to tenderness were [“fondness,” “sentiment,” “sensibility,” “warmth,” “gratitude,” “passion,” “feeling,” “ardor,” “friendship,” “affection”]. This suggests that tenderness was used in our corpus with the correct meaning. Our rule of thumb was to keep words for which more than 5 out of 10 most similar words were consistent with the intended concept underlying the bag of words. With this method, which has been used in previous studies (Jackson et al., 2019; Martins & Baumard, 2020), we could thus verify if the selected words were appropriate and eliminate inadequate terms. Crucially, while this process involves some subjectivity, the adequacy of the bag of words (as a whole) for the analysis is evaluated in the steps of internal and external validity (see next section).

In summary, with this procedure, we obtained search terms that reflected semantic and spatial proximity to “love” while obtaining distinct word sets for love’s emotional and physical components. Search term lists are depicted in Supplementary Tables S1 and S3.

### Internal and External Validation

After obtaining these ten bags of words, we computed their frequencies for each text and performed internal and external validation measures.

First, with this bag-of-words approach, it is difficult to determine whether certain words or dimensions are used in affirmative or negation contexts or whether these dimensions relate to the appropriate dimensions of love. To explore the dimensionality of the data, we performed a factor analysis with these ten categories (using the R function factanal(), from the “stats” package). For England, we only included in the analysis plays for which at least one word of each category was present. We eliminated outliers (|z-score|> 3) to avoid their disproportional influence in the data analysis. The final sample for England was comprised of 847 plays.

**Fig. 1:**
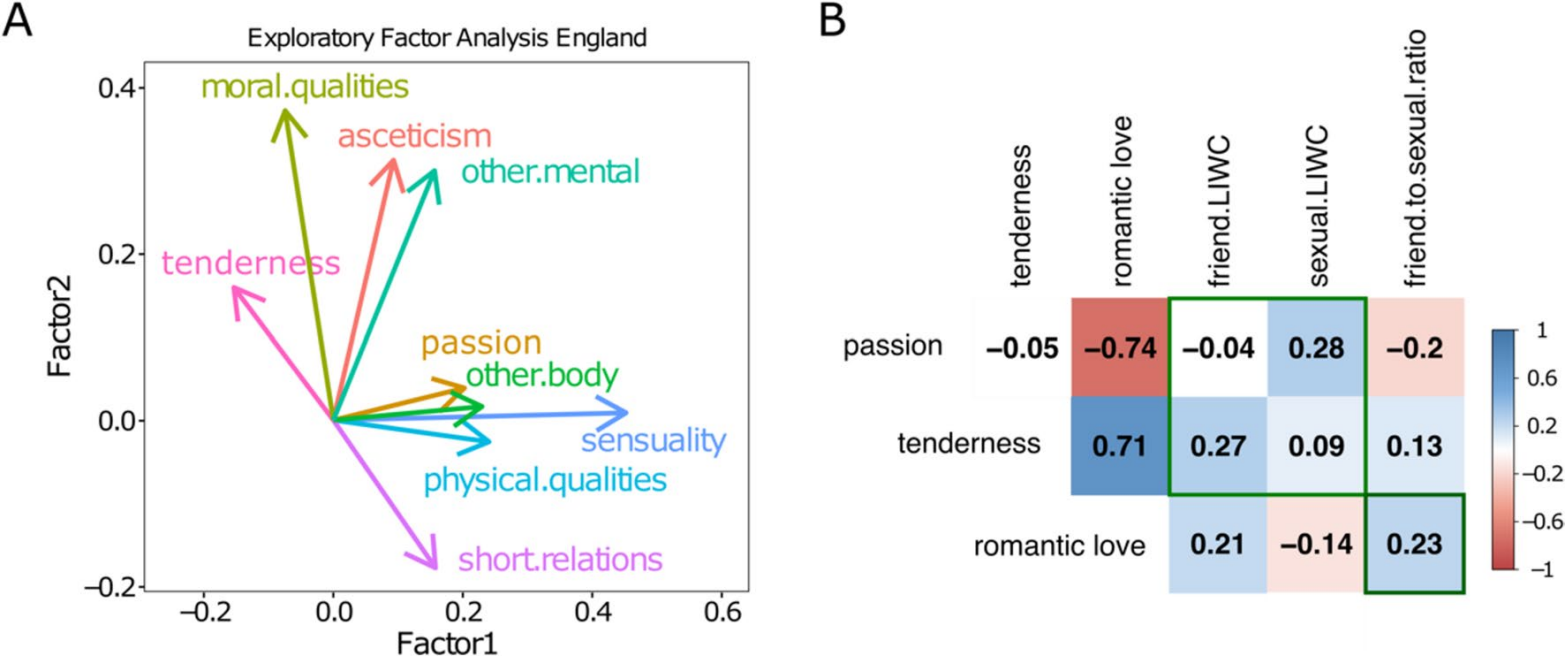
**a** Internal validation. Factor analysis with several variables related to emotional investment and desire. **b** External validation. Correlation with dimensions “Sexual” and “Friend” from the tool Linguistic Inquiry and Word Count (Pennebaker et al., 2015)

Crucially, the factor analysis demonstrated that the constructs of tenderness clustered together with other proxies of “emotional investment” (discipline, moral qualities, and mental traits), the constructs of passion clustered together with other proxies of desire (sensuality, physical qualities, and bodily traits) (Fig. 1) and that the two dimensions were orthogonal. This clustering structure suggests that our measure of tenderness is more likely to be used in the context of romantic love when contrasted with the measures of passion. Based on this analysis, we obtained a romantic love ratio for each play by subtracting the normalized (z-scored) passionate feelings from tender feelings:

$${\text{romantic}}\,{\text{love}}\, = \,Z\left( {{\text{frequency}}\,{\text{ tenderness}}} \right)\,{-}Z\left( {{\text{frequency}}\,{\text{ passion}}} \right).$$

A similar procedure was used in France. The frequency of love-related words was somewhat rarer for most categories; for example, with only 391 plays (out of 1030) containing words related to both categories of asceticism and pleasure, 774 plays contained words related to both tenderness and passion, and 762 plays contained words related to moral and physical qualities. To mimic our choice for England and to avoid further data loss, we choose the tenderness-to-passion ratio as our measure. After eliminating extreme outliers (|z-score|> 3), the final sample for France contained 762 plays.

To externally validate our measures of romantic love, we compared our ratios with other potential proxies of love from the tool Linguistic Inquiry and Word Count (LIWC), which is well validated for modern text (Pennebaker et al., 2015; Piolat et al., 2011). The LIWC is the gold standard tool for bags-of-word analysis. It is composed of 12 K words and is well validated for dozens of concepts by cross-referencing linguistic analysis of participants’ texts with their results on psychometric tests. The LIWC has been used in thousands of studies (e.g., Lanning et al., 2018; Vine et al., 2020) and has more than 10 K citations. More information on the validation processes can be found in the most recent tools’ manual (Boyd et al., 2022).

We chose the LIWC dimensions of family and friends as proxies of emotional investment and the sexual dimension as the proxy of passion. We computed the corresponding friends-to-sexual and family-to-sexual ratios. We found that our primary measure of romantic love was positively correlated with both proxy ratios from LIWC, both for England (friends-to-sexual *r* = 0.23, family-to-sexual *r* = 0.17) and France (friends-to-sexual *r* = 0.24, family-to-sexual *r* = 0.23); Supplementary Figures S2 and S5). As expected, these correlations are relatively weak since LIWC dimensions are not specifically designed for romantic love. However, they suggest our tenderness-to-passion ratio not only correctly distinguishes between those two dimensions of love but also does it in the correct direction, away from sexual-related language and toward words related to friendship and family.

### Analysis

To test how romantic love varied with time and the hypothesis that it increased with living standards, we ran linear mixed models with romantic love as the dependent variable, play author as the random factor, and year, GDP per capita (Broadberry et al., 2015; Ridolfi, 2017), wages (Humphries & Weisdorf, 2019), and life expectancy as predictors (Zijdeman & Ribeira da Silva, 2023). Importantly, these were not direct measures but estimates computed based on historical records. For example, wages are estimated from workers with annual pay (due to high fluctuations in daily pay), and GDPpc is estimated from agriculture, industry, and services outputs.

We computed the linear mixed models using the function lmer() with package lme4 (Bates et al., 2014). Best lambda transformations were found using boxcox() with package MASS (Venables & Ripley, 2013). Residual normality distribution was tested using Shapiro–Wilk (S–W) test. Models were reported using ANOVA (type = II) and the R package Anova() for *p *values. Pairwise differences were tested with emmeans() (Russell, 2018).

To test for the causality between romantic love and GDP per capita/life expectancy/wages, we ran cross-correlation (time-lag) analyses by first building a visualization with the function ccf() of the package tseries (Trapletti & Hornik, 2019). Second, we built models of romantic love at time T predicted by Year and by 21 additional terms corresponding to GDPpc/life expectancy with different time lags spanning the interval [T − 20, T + 20]. Then, we performed model selection using Bayesian Information Criterion (BIC), with the further constraint that the Year was in the final model (to control for long-term, secular trends). Finally, to control for temporal autocorrelations, we repeated the final model using generalized least squares (Pinheiro & Bates, 2000), and time (year) as the autoregressive component.

To test the effects of romantic love on pair bonding and child investment, we performed similar linear mixed models with nuptial rates and fertility rates (Wrigley et al., 1997) as dependent variables, time and romantic love as predictors, and author as the random factor. Again, these were historical reconstructions and not direct measures, as nuptial and birth rates were estimated from the records of four parishes.

All analyses were implemented in R-3.6.0.

## Results

### Proxies of Romantic Love Tap into Meaningful Constructs

Our measure of romantic love was chosen from a set of alternatives after an internal and external validation procedure described in Supplementary Materials. To ensure that the categories in the bags of words meaningfully captured the distinction between tender and passionate love, we performed a factor analysis with other emotional investment and desire measures. We found that several categories of long-term emotional investment (tenderness, moral qualities, discipline/asceticism, and other mental features) clustered together in a factor orthogonal to those categories of desire and attraction (passion, sensuality, physical qualities, sensuality, and other body traits) (Fig. 1a).

In addition to this internal consistency analysis, we evaluated whether our proxies were consistent with external (but much less sensitive) measures of love. We used the well-validated tool Linguistic Inquiry and Word Count (LIWC) (Pennebaker et al., 2015) to extract word frequencies related to the dimensions Sexual (including words like sex, breast, kiss, lust, naked, and passion, but also love and loved, etc.) and Friend (including boyfriend, girlfriend, fiancé, honey, lover and sweetheart, but also neighbor and comrade). These dimensions can be considered less specific proxies of desire and emotional investment. We found that tender feelings were well correlated with LIWC Friend, and passionate feelings were correlated with LIWC Sexual (Fig. 1b), suggesting that our measures are sensitive to the appropriate constructs.

Based on this analysis, we computed romantic love as the normalized difference between tenderness and passion (z(tenderness) − z(passion)) and used it for subsequent analyses (Fig. 1b).

One of the theoretical assumptions of our measure of romantic love is that it reflects some aspects of long-term thinking and future orientation. We used the LIWC measures of future and present orientation to test this assumption and calculated a Future-to-Present ratio. We found that our measure of romantic love was well predicted by both LIWC Friend-to-Sexual and Future-to-Present ratios when accounting for the time (years) [*β-year* = − 0.03, *95% CI* (− -0.06–0.10), *t*(160) = 0.42, *p* = 0.7; β*-future.to.present* = 0.19, *95% CI* (0.11–0.27), *t*(570) = 4.70, *p* < 0.001; *β-friend.to.sexual* = 0.28, *95% CI* (0.20–0.35), *t*(675) = 7.4, *p* < 0.001].

### Living Standards Predict the Importance of Romantic Love in the Early Modern Theater

**Fig. 2:**
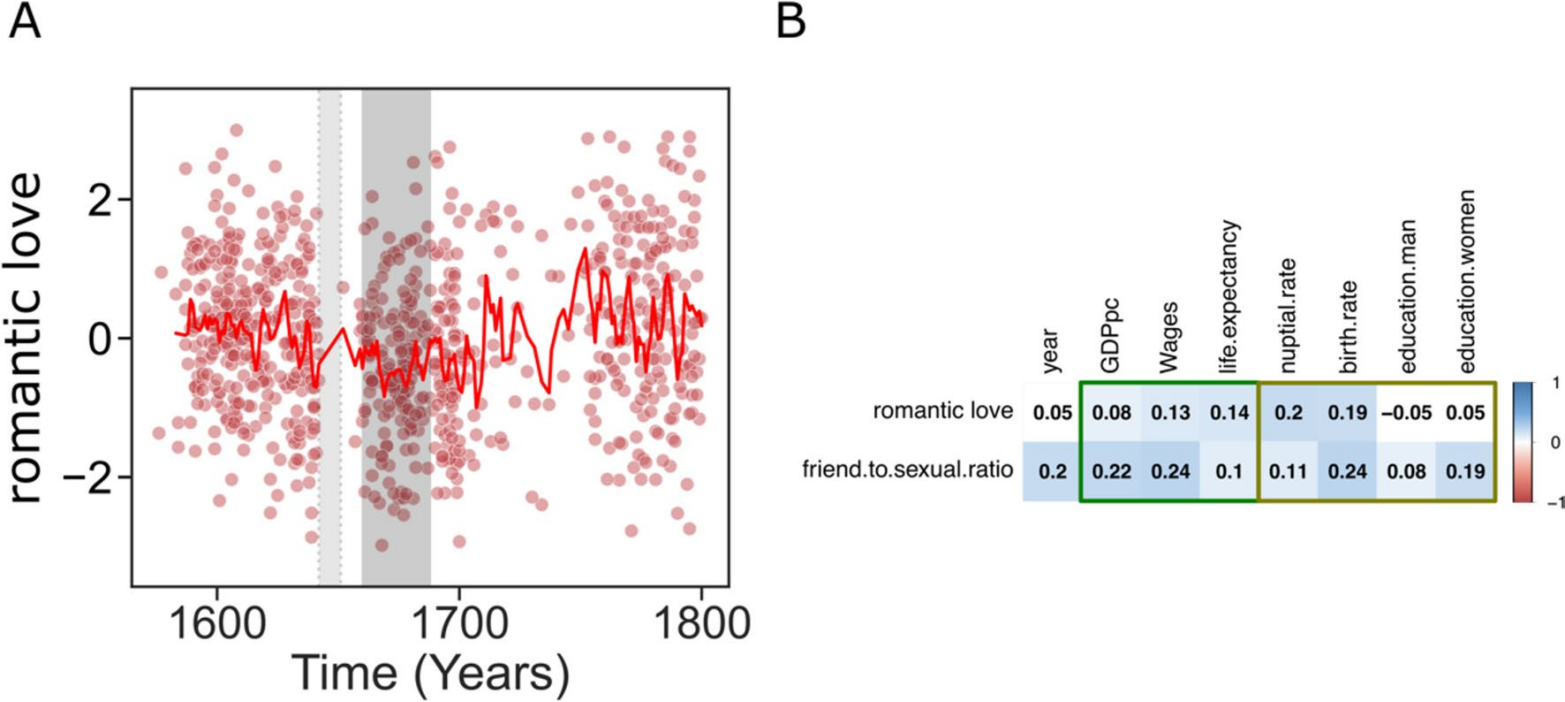
**a** Romantic love across the Early Modern period. Each red circle represents a theater play. The 3-year rolling average is represented as a red line. For historical reference, we depict the period corresponding to the English Civil War (light gray) and the Restoration (dark gray). **b** Correlation table between romantic love proxies and socioeconomic variables. The light green rectangle on the left denotes living standards variables, and the brown rectangle on the right denotes variables related to investment in partners and children. We depict both our romantic love ratio (tender-to-passionate feelings) and (for reference) the LIWC-based Friend-to-Sexual ratio

The variation of the specifically romantic components of love across the Early Modern period in England is depicted in Fig. 2a. Here, we depict two major political periods—The English Civil War and the Restoration—for temporal reference (light and dark gray areas, respectively). The salience of romantic love in theater seems to have decreased before the English Civil War and increased afterward (Separate tender and passionate feeling time series are depicted in Supplementary Figure S3).

Our first hypothesis (https://osf.io/wn6fs) was that living standards are related to the expression of romantic love in fiction. The correlation between romantic love and socioeconomic variables is depicted in Fig. 2b. Our proxy of romantic love was weakly but positively correlated with GDPpc [*r*(848) = 0.08, *p* = 0.02], wages [*r*(848) = 0.13, *p* < 0.001] and life expectancy at birth [*r*(848) = 0.14, *p* < 0.001]. A similar pattern was found for the Friend-to-Sexual ratio (Fig. 2b). To confirm the association between the importance of romantic love and standards of living, we ran linear mixed models (LMM) with author as random factor and time (years) as a covariate. Romantic love was well predicted by GDPpc [*β* = 0.24, *95% CI* (0.02–0.45), *t*(756) = 2.12, *p* = 0.033], wages [*β* = 0.23, *95% CI* (0.08–0.39), *t*(605) = 3.0, *p* = 0.003], and life expectancy [*β* = 0.10, *95% CI* (0.03–0.17), *t*(566) = 2.65, *p* = 0.008].

We also ran a model including all socioeconomic variables and found that the effect of wages was a significant predictor of romantic love [*β-year* = − 0.19, *95% CI* (0.44–0.05), *t*(623) = − 1.52, *p* = 0.13; *β*-wages = 0.17, *95% CI* (0.01–0.34), *t*(694) = 2.10, *p* = 0.036; *β-*GDPpc = 0.10, *95% CI* (− 0.14–0.33), *t*(777) = 0.81, *p* = 0.41; *β-life.expectancy* = 0.07, *95% CI* (− 0.01–0.14), *t*(662) = 1.75, *p* = 0.08]. This means that while all variables were growing within this period (potentially due to a third unknown variable), some variability was still explained by living standards (wages) when accounting for the shared variance between GDPpc, wages, and life expectancy, *and* controlling for the general linear growth of love across time (by adding *year* to the model).

To assess the temporal precedence between living standards and love, we performed a time-lag analysis (or lagged regression). Time lag analyses are a common tool to assess the causality between two time series, X and Y, by determining how well X at time T can be predicted by Y at different points in time, both before and after T (T − *n* and T + *n*, respectively) (Cromwell & Terraza, 1994). For instance, for this analysis, we built models of romantic love at time T predicted by year, GDPpc at time T, and 40 additional terms corresponding to GDPpc with different time lags spanning the interval [T − 20, T + 20], i.e., ranging from 20 years before to 20 years after the corresponding time point of love. First, we computed the full model containing all 41 GDPpc time lags. Then, we performed model comparison using BIC, which removed GDPpc lags stepwise until the best model was obtained. The only constraint was that the final model must include time (*year*) to control for the linear rise of love. Crucially, to prevent overestimation of GDPpc effects due to temporal autocorrelation, we computed the model using generalized least squares (GLS) (Pinheiro & Bates, 2000) with time (year) as first-order autocorrelation term. We performed similar time-lag analyses with wages and life expectancy.

**Fig. 3:**
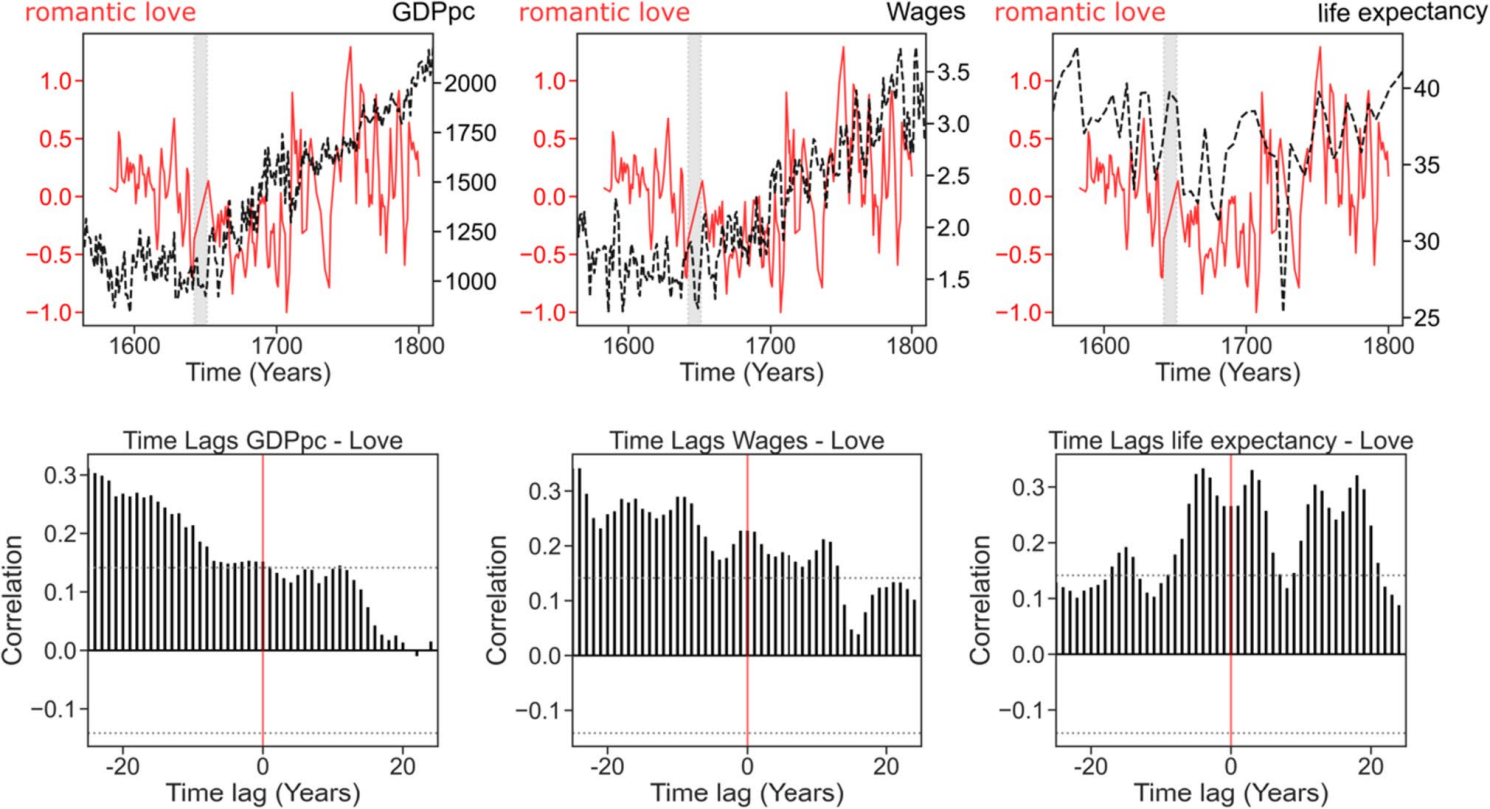
Love and Living standards (Top) Time series of love and socioeconomic variables. The red line depicts 3-year rolling average of love. The dashed dark line depicts socioeconomic data. (Bottom) Time lag analysis assessing the temporal precedence between sociodemographic variables and the love ratio using ccf() function (see methods)

First, regarding our living standards predictors of the importance of romantic love, we found that the variations of romantic love are clearly preceded by isomorphic variations of GDPpc and wages (Fig. 3).

The best model for love at time T (BIC = 513.9) included a positive prediction of GDPpc at time T−20 [*β* = 0.73, *95% *CI (0.45, 1.03), *t*(178) = 4.89, *p* < 0.001]. Similarly, love was positively predicted by wages (BIC = 492.8) at time T−9 [*β* = 0.54, *95% *CI (0.24, 0.83), *t*(168) = 3.59, *p* < 0.001]. In other words, when controlling for global linear trends and autocorrelations, love was positively predicted by the levels of GDPpc 20 years earlier and wages 9 years earlier but not by positive predictors after (Supplementary Table S2; Supplementary Figure S4 for model diagnostics).

In the case of life expectancy, the original data was linearly interpolated between data points separated by 5 years. To avoid extreme collinearities, we included in the initial lag analysis only time points separated by 5 years: T, T ± 5, T ± 10, T ± 15, and T ± 20. We ran four additional 5-year lag models with different starting points (T + 1, T + 2, T + 3, and T + 4) and did model selection using BIC. The best model (BIC = 523.9) included a positive predictor at time T−4 [*β* = 0.26, 95% CI (0.12, 0.41), *t*(178) = 3.53, *p* < 0.001].

### The Importance of Romantic Love in the Early Modern Theater Predicts Nuptial Rates and Births per Marriage

Our second set of hypotheses pertained to the relationship between romantic love and behavior. If the social importance of romantic love increases the tendency for long-term commitments, then nuptial rates will be higher in years with higher expressions of romantic love. Similarly, if romantic love increases investment in offspring, then we would expect it to be associated with lower birth rates, i.e., investing more resources in fewer offspring (Kaplan, 1996; Lawson & Mace, 2011). As depicted in Fig. 2B, we found a weak but positive correlation between romantic love and nuptial rates [*r*(847) = 0.20, *p* < 0.001], but also a positive correlation with birth rates [*r*(847) = 0.19, *p* = 0.007]. When accounting for the effects of time and the random effects of author, romantic love was still positively associated with nuptial rates [*β* = 0.20, *95% CI* (0.12–0.27), *t*(269) = 5.08, *p* < 0.001] and birth rates [*β* = 0.20, *95% CI* (0.11–0.29), *t*(256) = 4.41, *p* < 0.001].

These findings are mixed regarding our hypotheses that rising living standards and romantic love lead to higher pair bonding (marriage) and investment in each child (lower birth rates). On the one hand, nuptial rates increase with the expression of romantic love. However, birth rates display a similar pattern, against our predictions. One particular confound is that nuptial and birth rates are highly correlated (*r*(225) = 0.59, *p* < 0.001), meaning that more pair bonding leads to more births. Thus, after having preregistered, we realized that low birth rates might not be the optimal proxy to evaluate investment in each child. To disentangle the effects of nuptial and birth rates, we computed an exploratory birth-to-marriage ratio (BNR = birth rate/nuptial rate), which is higher if births rise faster than marriage and lower if marriage rates rise faster than births. When accounting for the effects of time and the random effects of author, there was a negative association between romantic love and the birth-to-marriage ratio [*β* = − 0.13, *95% CI* (− 0.22 to − 0.04), *t*(624) = − 2.87, *p* = 0.004]. Note that nuptial and birth rates are equally correlated with love. Hence, this finding for BNR is not caused by the fact that nuptial rates alone are driving the latter effect.

**Fig. 4:**
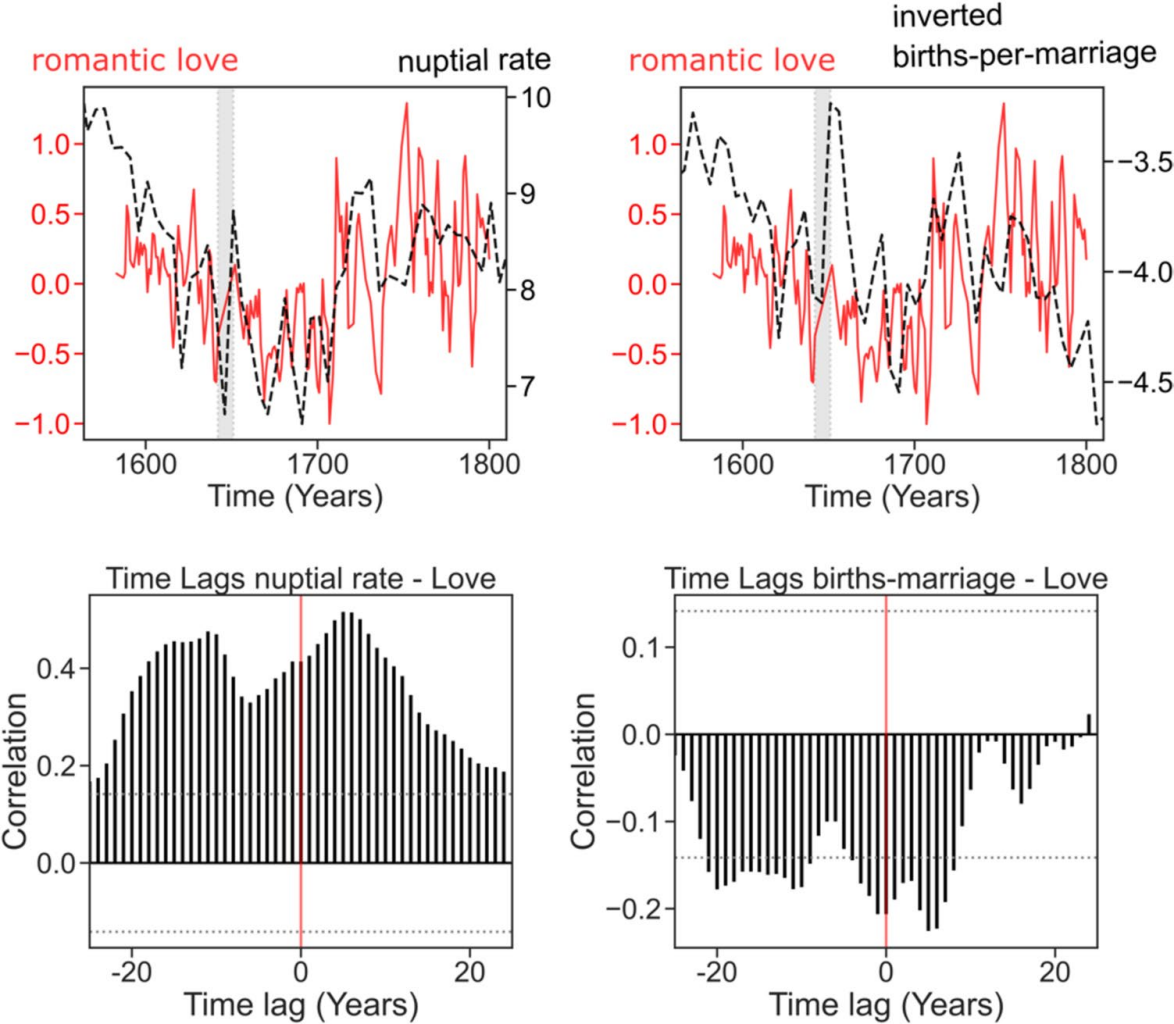
Love, pair bonding, and child investment (Top) Time series of love and sociodemographic variables. The red line depicts 3-year rolling average of love. The dashed dark line depicts sociodemographic data. (Bottom) Time lag analysis assessing the temporal precedence between sociodemographic variables and the love ratio using ccf() function (see methods)

To investigate whether the temporal relationship between love and outcomes is related to pair bonding and child investment, we performed lag analyses (Fig. 4). For nuptial rates and child per marriage, we used the same procedure as for life expectancy due to the data being generated by similar 5-year interpolations. We found that the best models of romantic love positively predicted nuptial rates [BIC = 516.5; *β* = 0.33, *95% CI* (0.19, 0.47), *t*(178) = 4.61, *p* < 0.001] and negatively predicted births per marriage [BIC*:* 526.2; *β* = − 0.27, *95% CI* (− 0.45, − 0.10), *t*(178) = − 3.11, *p* = 0.002] at year T + 6 (Supplementary Table S2, Supplementary Figure S4).

### Gross Domestic Product Variations Also Predict the Expression of Romantic Love in the French Early Modern Theater

Finally, we wanted to replicate our analysis with another major dramatic tradition, namely, French theater. Due to limitations in the availability of socioeconomic French data for this period, we limited our analysis to the relationship between GDPpc and love. We chose the same tenderness-to-passion ratio as for England (see bags of words in Supplementary Table S3), which was also correlated with the Friend-to-Sexual ratio from the French LIWC (Piolat et al., 2011) (*r*(762) = 0.22, *p* < 0.001) (Supplementary Figure S5).

**Fig. 5:**
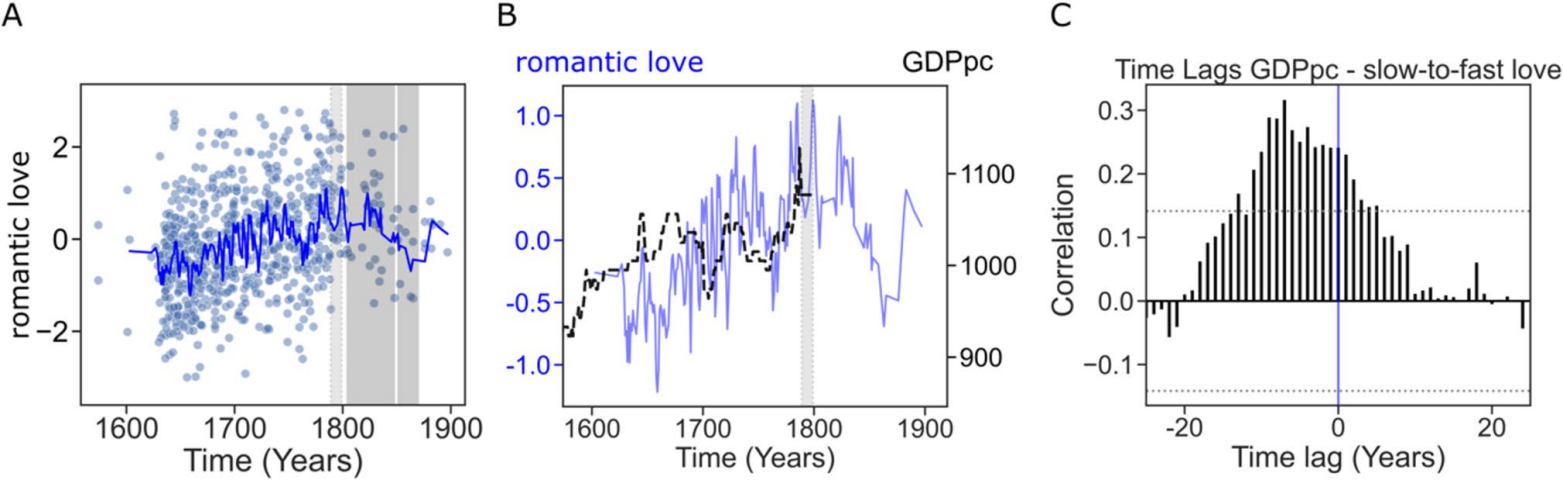
**a** Romantic love across the Early Modern period in France. Each blue circle represents a theater play. 3-year rolling average is represented as a blue line. For historical reference, we depict the period corresponding to the French Revolution (light gray). **b** Time series of love and GDPpc. The blue line depicts 3-year rolling average of love. The dashed dark line depicts GDPpc yearly average **c** Time lag analysis assessing the temporal precedence between GDPpc and the love ratio using ccf() function (see methods)

The temporal dynamics of love, GDP, and their relationship are depicted in Fig. 5 (Separate tender and passionate feeling time series are depicted in Supplementary Figure S6). First, we found that GDPpc was correlated to romantic love (*r*(708) = 0.157, *p* < 0.001). However, when we controlled for the random effects of author and for long-term linear trends (year as a covariate), the association between love and GDPpc was weaker [*β*_GDPpc_ = 0.07, *95% CI* (− 0.007 to 0.15), *t*(662) = 1.79 *p* = 0.07]. As for England, we performed a lag analysis. The best model for love at time T (BIC = 433.1) included a positive prediction of GDPpc at time T-9 [*β* = 0.80, *95% CI* (0.26, 1.31), *t*(160) = 2.90, *p* = 0.004], and no positive predictors afterward (Supplementary Table S4, Supplementary Figure S7). These results, together with the cross-correlation analysis (Fig. 5c), suggest that the variations of GDPpc precede isomorphic variations in the expression of romantic love when the effects of temporal autocorrelation and the global trends across time are accounted for. However, these lag results must be taken cautiously since there were also negative predictors both before and after time T (Supplementary Table S4), suggesting nonlinear effects.

## Discussion

In this paper, we sought to investigate the relationship between the evolution of living standards and the social importance of romantic love in the Early Modern Period. We measured the expression of romantic love in theater plays by developing a proxy that isolates the emotional investment components of romantic love (tenderness and affection) by contrasting them with passion and desire. Crucially, we measured the frequency of words about tender and passionate feelings when these were used in the spatial proximity (within a window of three nouns or adjectives) of seed words directly related to the concept of love (love, lover, beloved, etc.). Moreover, we performed internal and external validation procedures to ensure that our measure tapped into the appropriate constructs. Interestingly, our measure of romantic love was well predicted by measures of friendship and family vs. sexuality and of future vs. present orientation from LIWC. Also, our results align with qualitative historical surveys describing a rise in the importance of mutual affection and emotional attachment during the Early Modern Period (Coontz, 2006).

Using this novel tool, we found that the variation of proxies of living standards (GDPpc, wages, and life expectancy) generally (1) correlated with variations in the expression of romantic love and (2) temporally preceded those variations. The latter result was robust to the control of temporal autocorrelations and global linear trends. We partially replicated this finding for France (although we only had GDPpc as a proxy of living standards). These results converge with Baumard et al. (2022) but offer a much more fine-grained analysis of psychological constructs, temporal resolution, and economic data.

In addition to these main findings, we also explored the relationship between the expression of romantic love and behavioral changes related to the investment in partners and children. We proxied investment in partners with nuptial rates and investment in children with a decrease in birth. First, we found that nuptial rates increase in periods of higher expression of romantic love and that the variations of romantic love precede those of nuptial rates. Crucially, widows and widowers waited longer to remarry in the eighteenth century and did so less frequently than in the sixteenth and seventeenth centuries (Todd, 1994; Wrigley et al., 1997). Hence, these results are more likely to reflect the effects of the first wedding than remarriage. Second, we found that the salience of romantic love correlated positively with crude birth rates (against our hypothesis). However, when we corrected the crude birth rates by nuptial rates, we found that romantic love predicted a decrease in the number of births *per marriage*.

We found a similar correlational structure using the LIWC, but while its bags of words are relatively good proxies of romantic love (friendship/family vs sexual language), they are unspecific. First, our custom bags of words are specifically designed for the Early Modern Period, and they are measured relative to their use in the vicinity of the words such as “love,” “loving,” and “beloved.” Conversely, LIWC quantifies the frequencies of these words in the whole text. Thus, an increase in friendship-related words can be partially confounded by a general increase in prosociality, as described in previous work (Martins & Baumard, 2020). While having similar correlations from a completely different set of words is encouraging, these results should be interpreted cautiously.

These results align with recent advances in behavioral sciences, showing an association between high living standards, parental investment, and the social importance of romantic love (Baumard et al., 2022; Belsky et al., 1991; Chisholm et al., 2005; Levine et al., 1995; Quinlan, 2003, 2007; Quinlan & Quinlan, 2007; Raby et al., 2015; Schmitt, 2008). They suggest that the rise of romantic love observed by cultural historians could result from the rise of living standards in England and France during the Early Modern Period and partially explain the significant change in interpersonal relationships and family arrangements.

Finally, the generalizability of these findings presents some challenges. First, we present data mostly from England, and although we have a partial replication for France, the data availability and quality are much reduced. Second, these data pertain to two European countries with geographical and cultural affinities, and a larger scale study with independent geographical data points is necessary to strengthen the conclusions. For instance, it would be interesting to test whether the rise of affection and tenderness can also be observed in Chinese (Idema, 2001; Shih, 2015) and Japanese fiction (Keene, 1999) when these societies also changed romantic behavior (Lu, 2021; Pan, 2015; Wang, 1994), and if so, whether these trends are associated with economic development and behavioral change. Interestingly, a recent study analyzed Wikipedia summaries of fiction covering 19 geographical areas and spanning 3,800 years and found that the theme of love is more salient during periods of high affluence (Baumard et al., 2022), hinting that our findings might be generalizable. A recent cross-cultural study in 13 countries has shown that kissing is more common in countries where wealth inequality is higher, hinting that romantic behavior might be modulated by living standards and increased mate competition (Watkins et al., 2019). However, kissing is seen as a monitoring device for the quality of relationships, and it is unclear whether it directly relates to tenderness and emotional investment, as monitoring might increase when trust is low.

A third important point is that the content of theater plays reflects mostly the elites’ preferences, which might create noise in the association between romantic love and society-wide outcomes such as nuptial and birth rates. Importantly, while the values of social groups may differ on average, the crucial object of analysis is the diachronic variation that may be similar across social groups (and cultural media). This assumption is consistent with cultural analyses, suggesting that high-culture preferences often influenced the general zeitgeist in the Early Modern Period (Backscheider, 1993; Chiari & Laroque, 2017). This might be particularly the case in theater plays, which were attended by the elites, the middle classes, and the working classes (the “penny audience,” who paid a penny to stand close to the stage). Future studies targeting popular art forms might increase the signal-to-noise ratio, though they might be less available for the Early Modern Period. One could also test the relationship between the preferences of different social classes by contrasting the trends expressed in theater with even more elitist art forms such as opera and novels in the eighteenth century, or even paintings (as portraits of couples and families could communicate affection and love).

Another important point is whether the relationship between romantic love, birth rates per wedding, and living standards generalizes to the modern period, with access to modern contraception. We believe this is likely the case since countries undergo a demographic transition and a drop in fertility with rising living standards. Finally, while wars can negatively affect access to wealth and mates, this does not seem true in the aftermath of the English Civil War. While the post-war period could have affected nuptial rates, these seem to have decreased in the century before the war. Conversely, wages and GDPpc started a growth trajectory after the war, so the potentially deleterious effects of the latter do not seem to be long-lasting.

In conclusion, in this study, we developed and validated an instrument to measure the diachronic variations in the salience of romantic love in theater plays, a popular cultural medium throughout the Early Modern Period. We found that the expression of romantic love increased in the eighteenth century both in England and France when the living standards also started to rise, and it predicted the rise of long-term pair-bonding behavior. These interesting associations between material resources, psychology, and social behaviors align with recent developments in behavioral ecology. Whether they generalize to modern culture is an interesting topic of future research.

### Supplementary Information

Below is the link to the electronic supplementary material.

Supplementary file1 (DOCX 1747 kb)

## Data Availability

All datasets and scripts are available in the Open Science Forum repository https://osf.io/ka3th/?view_only=9d827995280c42a5ab89fa7cbcedac03.
