# Supplementary material for: Reproductive Strategies and Romantic Love in Early Modern Europe
Source: Arch Sex Behav. 2023 Dec 26;53(3):901–15. doi: 10.1007/s10508-023-02759-4 (PMC10920442; doi:10.1007/s10508-023-02759-4)
Supplement: Supplementary file 1 — Supplementary file1 (DOCX 1747 kb) [file 10508_2023_2759_MOESM1_ESM.docx]

## Supplementary Methods

**Building the Sets of Search Terms**

To generate the set of words associated with emotional investment/tenderness and attraction/desire, we used a combination of strategies.

First, we extracted the set of the 1000 most common nouns and adjectives occurring in the vicinity of love-related ‘seed’ words within a window of 3 words (before and after). For English, we used ‘love’, ‘lover’, ’beloved’, and ’loving’ as seeds. This step allowed us to capture the nouns and adjectives most frequently used within the ‘spatial’ context of love.

Second, we sorted words relevant to love and classified them according to ten broad categories, which could potentially be used to denote the emotional and physical components of love. The main categories were passionate feelings (e.g., passion, desire, jealousy) and tender feelings (e.g., tenderness, affection, fondness). However, we measured other relevant categories such as sensuality (e.g., pleasure, breast, bosom); discipline/asceticism (e.g., modest, chastity, innocence); physical qualities (e.g., young, handsome, beautiful); moral qualities (e.g., noble, virtue, judgment); short relationships (e.g., conquest, adventure, affair); long relationships (e.g., marriage, family, oath); other body parts (e.g., hand, face, eye); other mental parts (e.g., sound, mind, reason).

Third, to avoid selection bias, we used the word2vec algorithm (Mikolov et al., 2013) to check if the words within our selection had the desired meaning in the corpus. This algorithm automatically associates each word to a vector (a set of coordinates) in a high-dimensional space, which describes the semantic context in which each word is used. We trained a vector space with our own corpus, and thus with this tool we could verify if the selected words were appropriate and eliminate inadequate terms.

In summary, with this procedure, we obtained search terms that reflected both semantic and spatial proximity to the word ‘love’ while obtaining distinct word sets reflecting love's different components. Search term lists are depicted in Supplementary Table S1.

| **Proxy** | **Search Terms** |
| --- | --- |
| Tenderness | 'dear', 'kind', 'affection', 'care', 'tender', 'gentle', 'kindness', 'respect', 'safe', 'tenderness', 'mutual', 'safety', 'esteem', 'patience', 'fondness', 'compassion', 'confidence', 'calm', 'assurance', 'sincere', 'friendly', 'aid', 'admiration', 'careful', 'grateful', 'regard', 'secure', 'sympathy', 'harmony', 'trust', 'devotion', 'friendship' |
| Passion | 'passion', 'desire', 'fire', 'fool', 'flame', 'mad', 'jealous', 'blind', 'folly', 'foolish', 'haste', 'jealousy', 'wild', 'heat', 'jealousie', 'hot', 'rash', 'pang', 'passionate', 'eager', 'burn', 'impatient', 'ardent', 'doat', 'transport', 'temper' |
| Discipline/Asceticism | 'duty', 'pure', 'virgin', 'chaste', 'innocence', 'innocent', 'sacrifice', 'delay', 'chastity' |
| Sensuality | 'pleasure', 'breast', 'delight', 'bed', 'sex', 'kiss', 'bosom', 'breath', 'bliss', 'lust', 'venus', 'nymph', 'rapture', 'flesh', 'pleasant', 'sweetness', 'satisfaction', 'ass', 'satisfy', 'leisure' |
| Moral qualities | 'honour', 'virtue', 'noble', 'honest', 'sense', 'generous', 'virtuous', 'merit', 'honourable', 'vertue', 'gracious', 'value', 'honor', 'judgement', 'wisdom', 'honesty', 'character', 'nobl', 'resolve', 'brave', 'judgment', 'truth', 'frank', 'earnest', 'modest', 'modesty' |
| Physical qualities | 'fair', 'beauty', 'young', 'youth', 'charm', 'look', 'sight', 'pretty', 'charming', 'handsome', 'beauteous', 'swain', 'beautiful' |
| Long term relationship | 'true', 'faithful', 'oath', 'promise', 'constancy', 'constant', 'eternal', 'troth', 'faith', 'bond', 'everlasting', 'loyalty', 'future', 'loyal', 'couple', 'endless', 'union', 'real', 'wife', 'husband', 'vow', 'marriage', 'bride', 'altar', 'nuptial', 'ceremony', 'church', 'marry', 'family' |
| Short term relationship | 'mistress', 'wench', 'wanton', 'affair', 'quick', 'conquest', 'wretch', 'whore', 'fast', 'adventure', 'lover' |
| Other mental | 'heart', 'soul', 'mind', 'reason', 'thought', 'spirit', 'knowledge', 'brain', 'conscience' |
| Other body | 'eye', 'hand', 'face', 'body', 'head', 'ear', 'smile', 'foot', 'lip', 'knee', 'cheek', 'pale', 'hair', 'nose', 'tooth', 'finger', 'neck', 'mouth' |

**Table S1. Bags of words – England.** Classification of words used in the spatial vicinity of ‘love’, ‘lover’, ’beloved’ and ‘loving’, into categories potentially pertaining to emotional investment (tenderness, discipline/asceticism, moral qualities, long term relationships and other mental) and desire/attraction (passion, sensuality, physical qualities, short term relationships and other body).

**Internal validity of the measures of tender and passionate feelings**

The categories in Supplementary Table S1 were selected due to their potential relation to emotional investment (tenderness, discipline/asceticism, moral qualities, long-term relationships, and mental traits) and desire (passion, sensuality, physical qualities, short-term relationships, and body parts). We first computed the frequency of words related to each category for each text.

However, with this bag-of-words approach, it is difficult to determine whether certain words or dimensions are used in affirmative or negation contexts or whether they relate to emotional investment or desire. To explore the dimensionality of the data, we performed a Factor Analysis with these ten categories (using the R function factanal, from the ‘stats’ package). We included in the analysis only plays where love was mentioned and eliminated outliers (with z-score above 3 and below -3) to avoid disproportional influence in the data analysis. The final sample was comprised of 847 plays.

The acceleration factor suggested using 2 factors. We standardized the data and ran the Factor Analysis with varimax rotation. Scores of each variable projected onto the 2-dimensional space are depicted in Figure 1. Variables relating to emotional investment seem to load higher in Factor 2, while variables about desire seem to load higher in Factor 1. The only exception is the variable ‘long long-term relationships’, which includes many words related to marriage, both the institution and the ceremony. To obtain a cleaner pair of factors, we repeated the factor analysis excluding this variable. The results suggest a reasonable separation of emotional investment into Factor 1 and desire into Factor 2. We computed 4 specific ratios: one based on our primary dichotomy of tenderness vs. passion (feelings ratio) and two others based on moral-to-physical qualities (traits ratio) and asceticism-to-sensuality (behavior ratio). The final ratio included all slow vs. fast love categories.


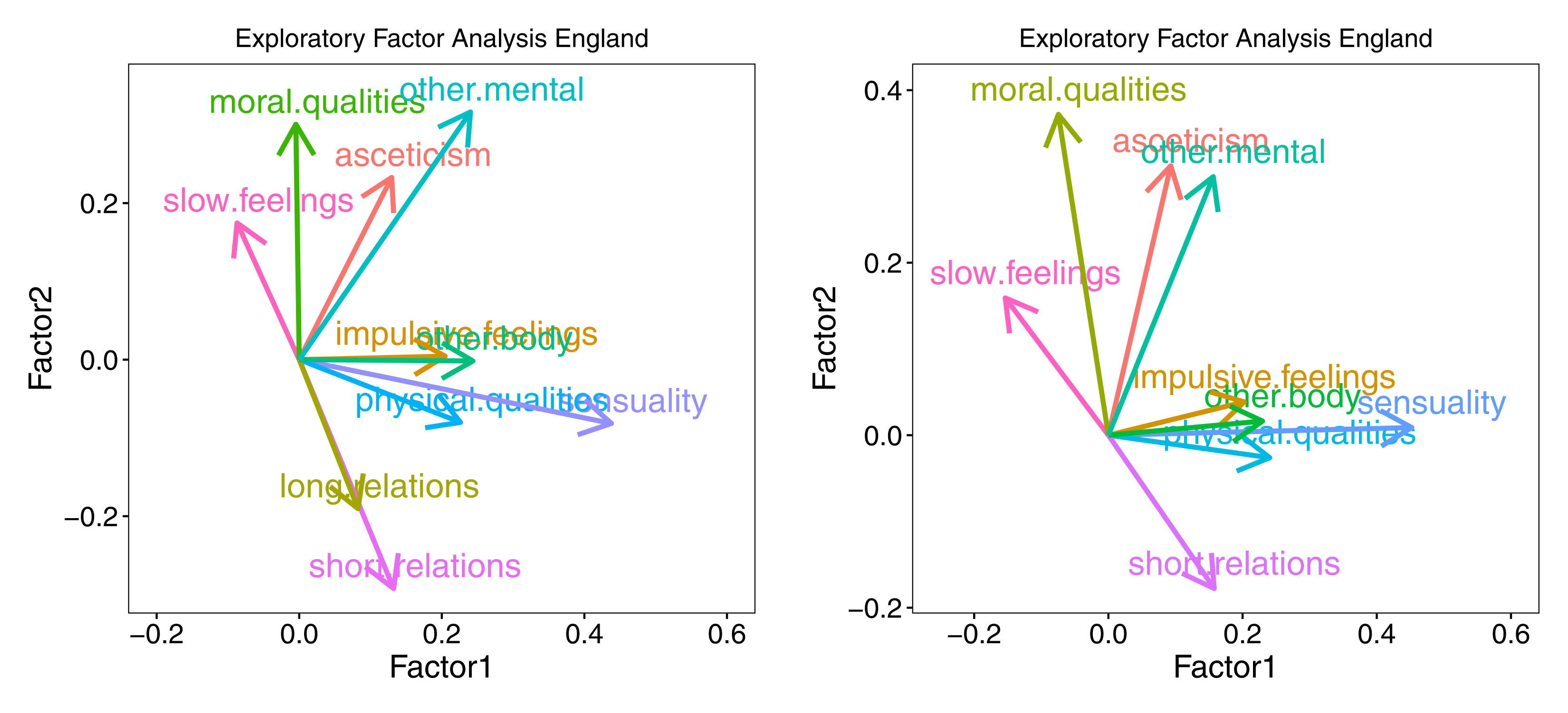


**Figure S1.** **Factor Analysis including ten variables (left).** Five potentially related to emotional investment (tenderness/slow feelings, discipline/asceticism, moral qualities, long-term relationships, and mental traits), and five potentially related to desire (passion/fast feelings, sensuality, physical qualities, short-term relationships, and body parts. The analysis shows that emotional investment variables load higher in Factor 2, while variables related to desire load higher in Factor 1. The only exception is long-term relationships, which load higher on Factor 1 and negatively on Factor 2. We repeated the analysis without the latter variable (right).

**External validation – LIWC**

To externally validate our measures of romantic love, we compared our ratios with other potential proxies of romantic love from the tool Linguistic Inquiry and Word Count (LIWC), which is well validated for modern text (Pennebaker et al., 2015). We chose the LIWC dimensions of family and friend as proxies of emotional investment and the sexual dimension as the proxy of desire and computed the corresponding Family-to-Sexual and Friend-to-Sexual ratios. We found that the global ratio was weakly (though significantly) correlated with the Family-to-Sexual and Friend-to-Sexual ratios, while this correlation was even weaker for the traits and behavior ratios (Figure S2). Conversely, the primary measure of tender-to-passionate feelings was moderately well-correlated with both proxy ratios from LIWC.

Taking the internal and external validation procedures together, we conclude that within this dataset, the tender-to-passionate feelings ratio (our primary measure) is more likely to be the best proxy of romantic love. Crucially, the behavior and trait ratios (about the contrasts asceticism-to-sensuality and moral-to-physical qualities, respectively) seem orthogonal to similar proxies in LIWC.


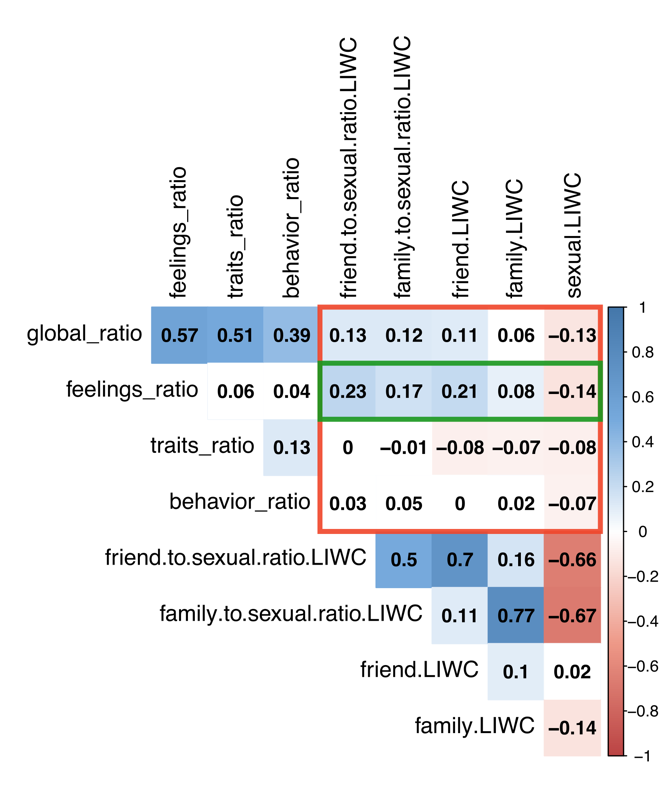


**Figure S2.** **Correlation table between our custom proxies of love (global, feelings, behavior, and traits) and similar proxies from the LIWC.** The proxy of tender-to-passionate feelings seems the closest to similar dimensions from the LIWC and thus is preferred for subsequent analyses.

**
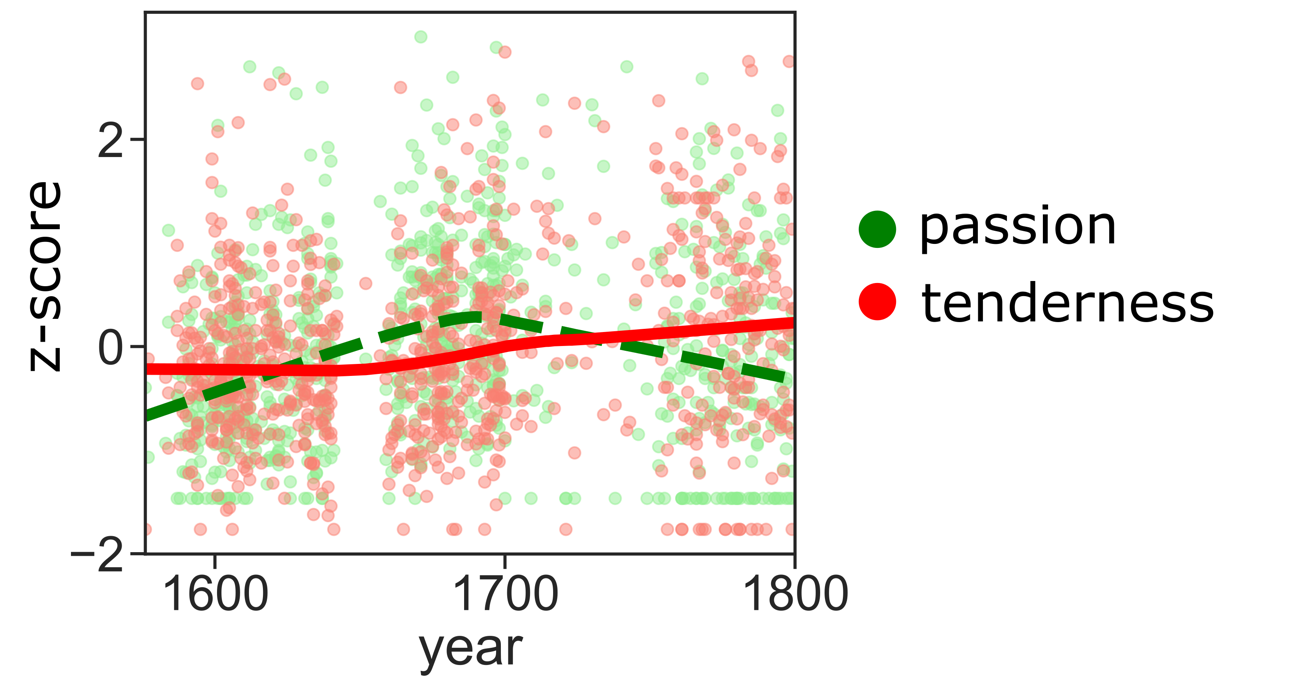
Figure S3. Time series of tender (red) and passionate (green) feelings in the English early modern period.**

| **love lags** | | | | | |
| --- | --- | --- | --- | --- | --- |
|  | | | | | |
|  | Dependent variable: | | | | |
|  |  | | | | |
|  | Love | | | | |
|  | (1) | (2) | (3) | (4) | (5) |
|  | | | | | |
| year | -0.547^***^ | -0.351^**^ | 0.120 | 0.053 | 0.246^***^ |
|  | (0.150) | (0.148) | (0.075) | (0.071) | (0.089) |
|  |  |  |  |  |  |
| GDP T-20 | 0.733^***^ |  |  |  |  |
|  | (0.150) |  |  |  |  |
|  |  |  |  |  |  |
| Wages T-9 |  | 0.540^***^ |  |  |  |
|  |  | (0.150) |  |  |  |
|  |  |  |  |  |  |
| life expectancy T-4 |  |  | 0.263^***^ |  |  |
|  |  |  | (0.075) |  |  |
|  |  |  |  |  |  |
| Nuptial rate T-6 |  |  |  | 0.327^***^ |  |
|  |  |  |  | (0.071) |  |
|  |  |  |  |  |  |
| Birth/Nuptial rate T+6 |  |  |  |  | -0.274^***^ |
|  |  |  |  |  | (0.088) |
|  |  |  |  |  |  |
| Constant | 0.002 | 0.020 | -0.001 | 0.0002 | -0.002 |
|  | (0.070) | (0.082) | (0.075) | (0.070) | (0.075) |
|  |  |  |  |  |  |
|  | | | | | |
| Observations | 181 | 171 | 181 | 181 | 181 |
| Log Likelihood | -243.951 | -233.536 | -249.000 | -245.251 | -250.113 |
| Akaike Inf. Crit. | 497.902 | 477.071 | 508.000 | 500.503 | 510.226 |
| Bayesian Inf. Crit. | 513.895 | 492.780 | 523.992 | 516.495 | 526.218 |
|  | | | | | |
| Note: | ^*^p^**^p^***^p<0.01 | | | | |

**Table S2. Lag analyses for the English data.** (1) GDPpc (per capita Gross Domestic Product) model (2) Wages model (3) Life Expectancy model (4) Nuptial rates model (5) Births per marriage model.

##
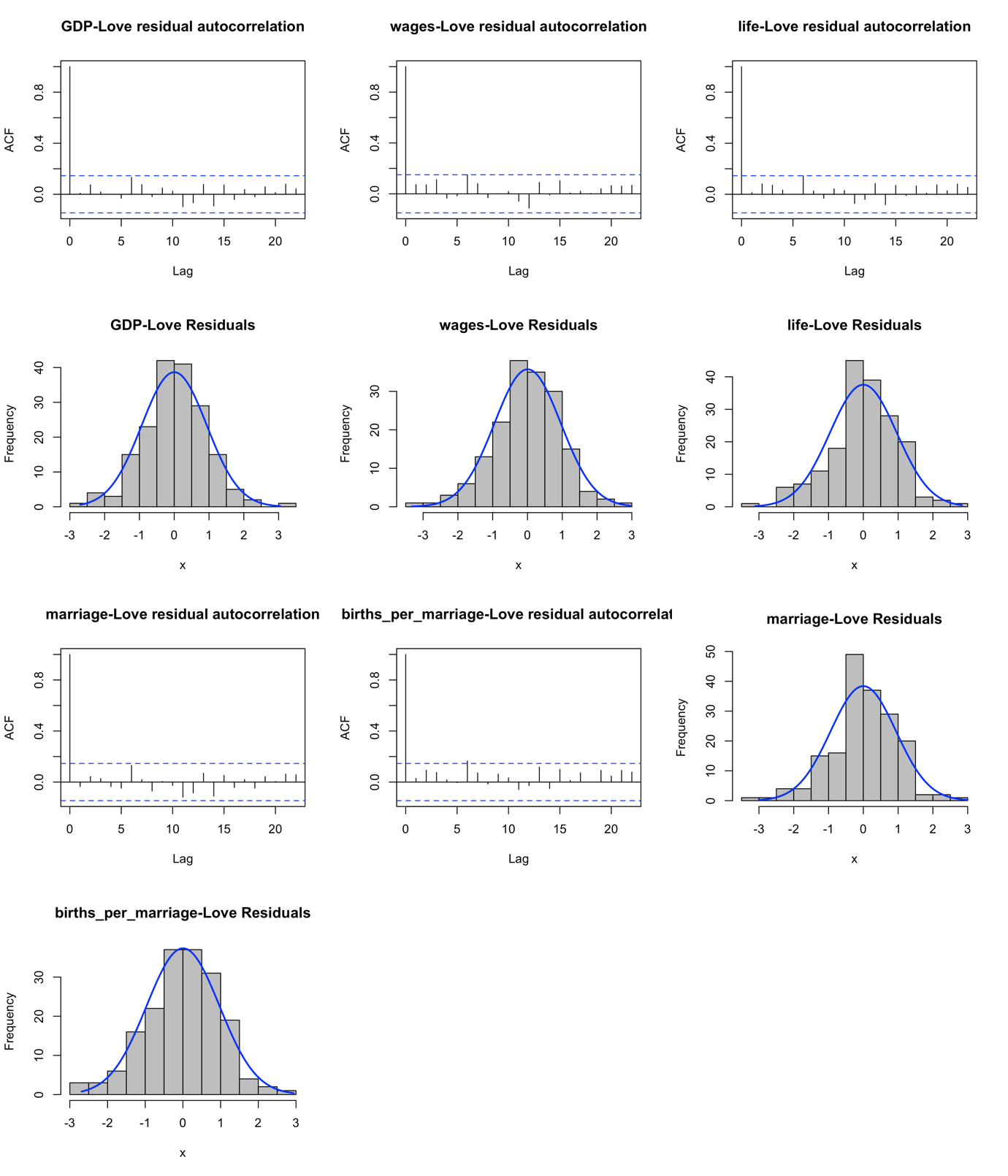


**Figure S4. Diagnostics for Lag analyses for the English data:** All models have normal residuals and in general low autocorrelations (ACF).

## Replication with French Theatre

| **Proxy** | **Search Terms** |
| --- | --- |
| Tenderness | affection', 'amitié', 'assurance', 'attachement', 'bonté', 'complaisance', 'complaisant', ‘confiance', 'considération', 'constance', 'constant', 'courtoisie', 'estime', 'fidèle', 'fidélité', ‘franchise', 'fraternel', 'patience', 'précaution', 'reconnaissance', 'reconnaissant', 'sensible', 'sincere', 'tendre', 'tendresse', 'véritable' |
| Passion | 'amorce', 'appât', 'appas', 'ardent', 'ardeur', 'audacieux', 'aveugle', 'aveuglement','avide', 'attrait', 'bouillant', 'brûlant', 'caprice', 'chaleur', 'désir', ‘effronté', 'emportement', 'empressement', 'envieux', 'extravagance', 'extravagant', 'feu', ‘flambeau', 'flamme', 'folie', 'fou', 'fureur', 'furie', 'impatience', 'imprudence', 'imprudent', ‘impétueux', 'inconstance', 'indomptable', 'insensé', 'jalousie', 'jaloux', 'libertin', 'légèreté', ‘manie', 'obstination', 'obstiné', 'passion', 'rebelle', 'sot', 'sottise', 'tempêteur', ‘téméraire', témérité', 'véhément', 'égarement', 'étourderie' |
| Discipline/Asceticism | 'chaste', 'devoir', 'innocence','pudeur', 'pudique', 'pur', 'sacrifice', 'timidité', 'épurer' |
| Sensuality | 'allégresse', 'amusement', 'bacchus', 'baise', ‘baiser', 'contentement', 'délice', 'fraîcheur', 'ivresse', 'jeunesse', ‘joie', 'jouissance', 'lit', 'nymphe', 'parfum', 'plaisir', 'satisfaction', 'volupté' |
| Moral qualities | 'brave', 'candeur', 'civilité', 'considérable', 'courage', 'courageux', 'dignité', ‘estimable', 'fermeté', 'généreux', 'générosité', 'habile', 'honneur', 'honnête', 'humanité', ‘humilité' 'jugement', 'louable', 'modeste', 'modestie', 'mérite', 'noble', 'persévérance', ‘politesse', 'prudence', 'prudent', 'raisonnable', 'respectable', 'respectueux', 'sage', 'sagesse', 'savant','sensé', 'sincérité', 'talent', 'timide', 'vaillance', 'valeur', 'vertu', 'vertueux', 'vigueur' |
| Physical qualities | 'adorable', 'agréable', 'aimable', 'beau', 'beauté', 'belle', 'charmant', 'charme', ‘jeune', 'jeunesse', 'joli', 'merveilleux', 'mignon', 'parfaire', 'ravissant' |
| Long term relationship | ‘main’,'époux', ‘épouse', ‘fidèle', ‘mari', ‘mariage', ‘serment', ‘éternel', ‘légitime', ‘famille', ‘épouse', ‘fidélité', ‘promesse', ‘union', ‘sacré', ‘engagement', ‘noce', ‘ménage', ‘vœu', ‘autel', ‘lien', 'foi' |
| Short term relationship | ‘infidèle', ‘liberté', ‘aventure', ‘libre', ‘volage', ‘conquête', ‘inconstance', ‘inconstant', ‘infidélité', ‘frivole', ‘vice', ‘amant', 'maîtresse' |
| Other mental | 'âme', 'cœur', 'esprit', 'connaissance' |
| Other body | 'corps', 'bouche', 'bras', 'visage', 'genou', 'main', 'tête', 'pied', 'genou', 'oreille' |

**Table S3. Bags of words – France.** Classification of words used in the spatial vicinity of ‘amour’, ’amant’ and ‘aimer’ into categories potentially pertaining to emotional investment (tenderness, discipline/asceticism, moral qualities, long term relationships and other mental) and desire (passion, sensuality, physical qualities, short term relationships and other body).

## Internal and external validation

## For the French data, we repeated the external and internal validation procedure that we also used for England. For internal validation, we used an Exploratory Factor Analysis with all 10 proxies of love (Figure S4, left), and for external validation, we checked how our proxy ratios correlated with similar measures from the widely used text analysis tool LIWC (Figure S4, right). Based on this analysis (see Figure S4) and in parity with the English data, we also selected the tender-to-passionate feelings ratio as a proxy of Romantic love.

##
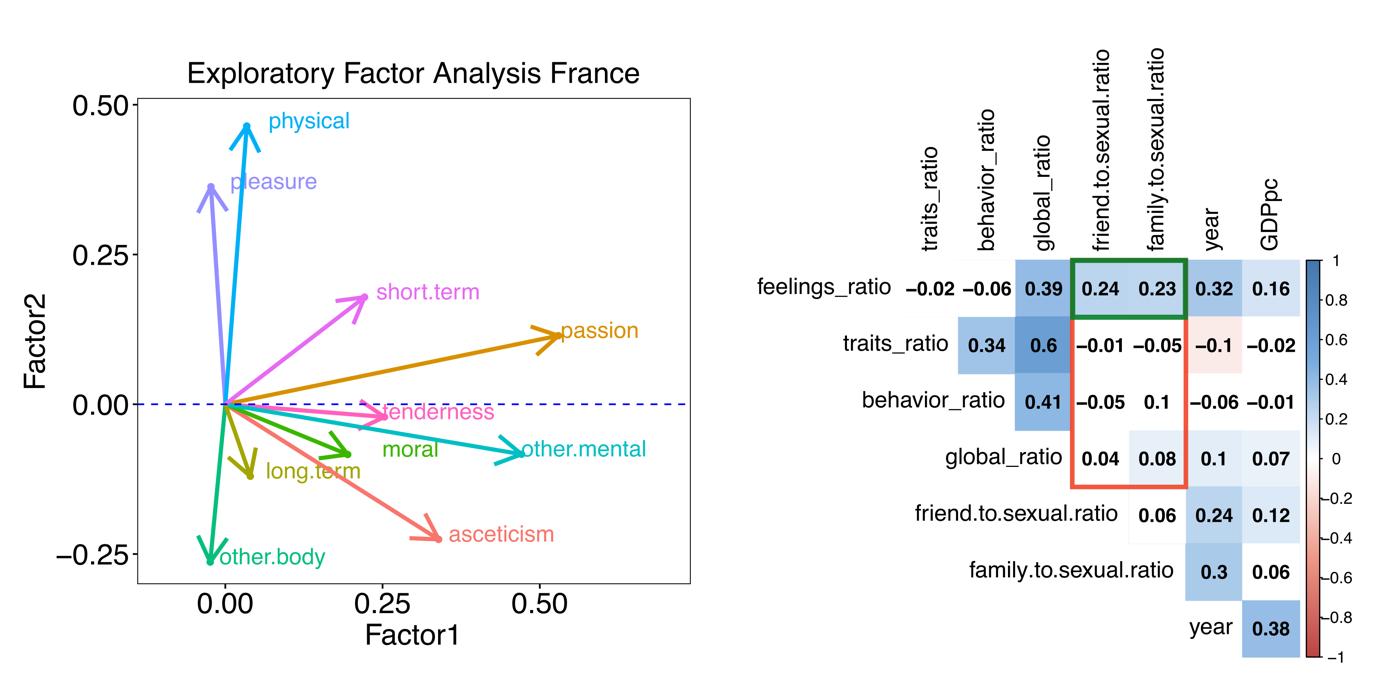


**Figure S5. Validation procedures for the French tender-to-passionate love ratio.** (**Left) Internal validation.** We ran an Exploratory Factor Analysis with the 10 proxies of emotional investment and desire and plotted the loadings along the two main factors. Factor 2 seems to distinguish between emotional investment (with negative loadings) and desire (positive loadings). Factor 1 is somewhat more ambiguous. (**right) External validation.** As for England, the tender-to-passionate feelings ratio seems to correlate with the LIWC ‘friend to sexual’ and ‘family to sexual ratios’ better than the alternative proxies.

## Time series - France

**
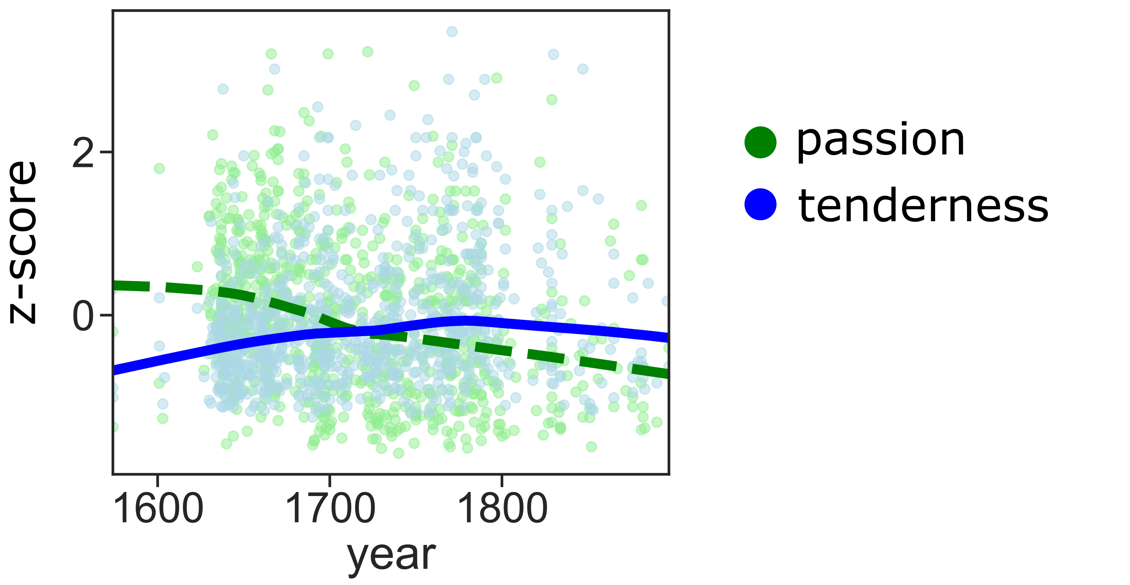
**

**Figure S6. Time series of tender (blue) and passionate (green) feelings in the French early modern period.**

## Lag analysis - France

| **love lags** | |
| --- | --- |
|  | |
|  | Dependent variable: |
|  |  |
|  | Love |
|  | |
| year | 0.840^***^ |
|  | (0.116) |
|  |  |
| GDPpc T-9 | 0.790^***^ |
|  | (0.268) |
|  |  |
| GDPpc T-10 | -0.804^***^ |
|  | (0.267) |
|  |  |
| GDPpc T+10 | -0.171^**^ |
|  | (0.073) |
|  |  |
| Constant | 0.397^***^ |
|  | (0.085) |
|  |  |
|  | |
| Observations | 160 |
| Log Likelihood | -198.796 |
| Akaike Inf. Crit. | 411.592 |
| Bayesian Inf. Crit. | 433.118 |
|  | |
| Note: | ^*^p^**^p^***^p<0.01 |

**Table S4. Lag analysis for the French data.** GDPpc (per capita Gross Domestic Product).

##
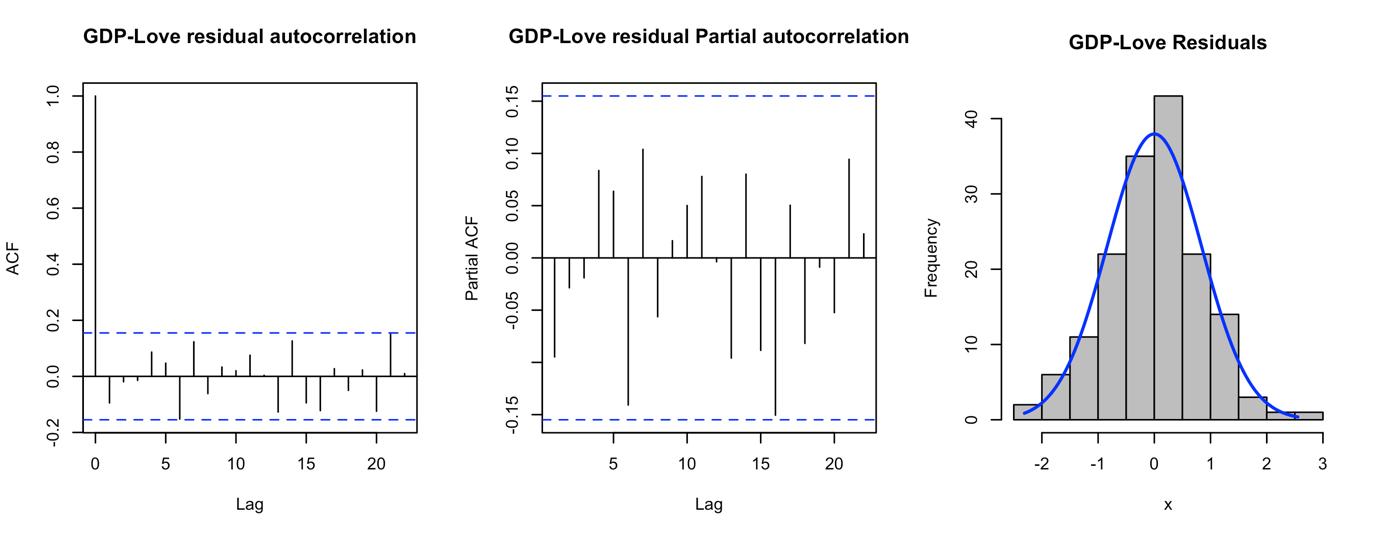


**Figure S7. Diagnostics Lag analyses (Lower row) for the French data:** Best lag analysis model had normal residuals and low autocorrelations (ACF).

## References

Mikolov, T., Chen, K., Corrado, G., & Dean, J. (2013). Efficient Estimation of Word Representations in Vector Space. *arXiv Preprint*, *arXiv:1301*, 1–12.

Pennebaker, J. W., Boyd, R. L., Jordan, K., & Blackburn, K. (2015). *The Development and Psychometric Properties of LIWC2015*. https://repositories.lib.utexas.edu/handle/2152/31333
